# Integrating Network Pharmacology and Metabolomics Study on Anti-rheumatic Mechanisms and Antagonistic Effects Against Methotrexate-Induced Toxicity of Qing-Luo-Yin

**DOI:** 10.3389/fphar.2018.01472

**Published:** 2018-12-18

**Authors:** Jian Zuo, Xin Wang, Yang Liu, Jing Ye, Qingfei Liu, Yan Li, Shao Li

**Affiliations:** ^1^Yijishan Hospital of Wannan Medical College, Wuhu, China; ^2^MOE Key Laboratory of Bioinformatics, Bioinformatics Division and Center for Synthetic and Systems Biology, Center for TCM-X, BNRist, Department of Automation, Tsinghua University, Beijing, China; ^3^School of Pharmaceutical Sciences, Tsinghua University, Beijing, China

**Keywords:** Qing-Luo-Yin, rheumatoid arthritis, network pharmacology, metabolomics, nucleotide metabolism

## Abstract

Qing-Luo-Yin (QLY) is a traditional Chinese medicine (TCM) formula used to treat Hot Syndrome-related rheumatoid arthritis (RA). Previously, we uncovered partial mechanisms involved in the therapeutic actions of QLY on RA. In this study, we further elucidated its anti-rheumatic mechanisms and investigated its possible interactions with methotrexate (MTX) *in vivo* using an integrating strategy coupled with network pharmacology and metabolomics techniques. Chemical composition of QLY was characterized by HPLC analysis. Collagen induced arthritis (CIA) was developed in male SD rats. The CIA rats were then assigned into different groups, and received QLY, MTX or QLY+MTX treatments according to the pre-arrangement. Therapeutic effects of QLY and its possible interactions with MTX *in vivo* were evaluated by clinical parameters, digital radiography assessment, histological/immunohistochemical examination, and serological biomarkers. Mechanisms underlying these actions were deciphered with network pharmacology methods, and further validated by metabolomics clues based on UPLC-Q-TOF/MS analysis of urines. Experimental evidences demonstrated that QLY notably alleviated the severity of CIA and protected joints from destruction. Re-balanced levels of hemoglobin and alanine transaminase in serum indicated reduced MTX-induced hepatic injury and myelosuppression under the co-treatment of QLY. Network-based target prediction found dozens of RA related proteins as potential targets of QLY. Upon the further biological function enrichment analysis, we found that a large amount of them were involved in nucleotide metabolism and immune functions. Metabolomics analysis showed that QLY restored amino acids, fatty acids, and energy metabolisms in CIA rats, which solidly supported its therapeutic effects on CIA. Consistently to findings from network pharmacology analysis, metabolomics study also found altered purine, pyrimidine, and pentose phosphate metabolisms in CIA rats receiving QLY treatment. All these clues suggested that inhibition on nucleic acid synthesis was essential to the immunosuppressive activity of QLY *in vivo*, and could contribute great importance to its therapeutic effects on CIA. Additionally, QLY induced significant antifolate resistance in rats, which would prevent folate from depletion during long-term MTX treatment, and should account for reduced side effects in combination regimen with MTX and QLY.

## Introduction

Rheumatoid arthritis (RA) is a prevalent systematic autoimmune disease, which is characterized by severe joint destruction and chronic local inflammation (Smolen and Aletaha, 2009). Conventional treatment approaches for RA patients are mainly dependent on disease-modifying anti-rheumatic drugs (DMARDs) (Tanaka, 2016). In the past decades, methotrexate (MTX) has become the anchor DMARD, and is extensively adopted as first-line regimen for the superior efficacy and economical merits (Cronstein, 2005). It significantly slows the progress of disease, and minimizes the detrimental effects on joints. However, there are a number of patients with inadequate response to it. Furthermore, despite the fact that low dose of MTX is well tolerated, the treatment usually accompanies with remarkable side effects (Dubey et al., 2016).

Traditional Chinese medicine (TCM) as one of the oldest continuously surviving traditional medicines is derived from clinical practices in ancient China. Much different from Western medicine, TCM emphasizes the integrality of body (Feng et al., 2006), and aims at multiple pathogenesis factors simultaneously upon the exact diagnosis of *Zheng* (a term in TCM, generally encompasses etiology, pathology and disease location) (Yu et al., 2006; Su et al., 2012). Contrarily, Western medicine usually modulates one single defined target or symptom based on the methodology of reductionism. By the development of system biology, limits of this strategy are gradually disclosed. Western treatments usually fail to achieve effective and sustainable outcomes in systematic and chronic diseases, and accompany with high risk of side effects (Feng et al., 2006; Yu et al., 2006). By comparison, TCM exhibits notable merits of efficacy and safety in many cases, and provides us an efficient alternative for treatments of some complicated diseases, such as RA. Objectively speaking, both the medical systems have their own advantages and shortcomings, and the integrated therapeutic approach would be more effective and safer when well optimized.

Traditional Chinese medicine formulas are usually composed of many herbs, which results in the complex chemical composition. Hence, it is difficult to fully understand their therapeutic mechanisms. High throughput techniques give us helpful tools to screen out potential bioactive ingredients from them. However, the dilemma is still hard to be resolved, for the formula should be taken as a whole but not the sum of some well investigated natural compounds. This situation makes network-oriented approaches more preferable (Li, 2015). To achieve a comprehensive and systematic understanding of their therapeutic mechanisms, new research strategies based on computational simulation and prediction flourish recent years (Guo et al., 2017). As one of these newly developed disciplines, network pharmacology possesses obvious advantages over conventional methods in elucidation of comprehensive mechanisms (Li et al., 2007; Hopkins, 2008). The network target-based concept could largely reflect complicated interactions between biomacromolecules and chemical ingredients. The research technique constructed under this guideline is regarded as a representative method of emerging network pharmacology (Barabasi et al., 2011), and has been successfully applied in many TCM related research fields, such as therapeutic mechanisms elucidation (Zhang Y. et al., 2015; Zhang et al., 2016), new pharmacological actions prediction (Zhang et al., 2018) and potential toxic ingredients screening (Zhang B. et al., 2015). Meanwhile, metabolomics focuses on systematic analysis of metabolites from drugs treated objects. It gives us an opportunity to obtain the panoramic view of network effects of formulas on bodies and testify results generated by computational modeling analysis.

Xin’an medical family is an important TCM academic school originated in South Anhui district in Song dynasty, and Zhang-Yi-Tie is one of its main branches still flourishing nowadays. Qing-Luo-Yin (QLY) is widely regarded as the representative formula of this sect, which was created by its 14th generation Jiren Li, a famous contemporary TCM master based on Xin’an medical theory and his medical experiences. It is composed of four components: Kushen (radix of *Sophora flavescens* Ait.), Qingfengteng [caulis of *Sinomenium acutum* (Thunb.) Rehder and E. H. Wilson], Huangbai (cortex of *Phellodendron chinense* C. K. Schneid.), and Bixie [rhizome of *Dioscorea collettii* var. *hypoglauca* (Palib.) S. J. Pei and C. T. Ting]. As a Cold natured formula, it is mainly used to treat the Hot Syndrome-related RA. Previously, we revealed that targets of QLY against RA-related key biological processes mainly involved in angiogenesis, inflammatory response and immune functions by using a network target-based research technique, but critical upstream factors leading to these changes were still unknown (Zhang et al., 2013). In the present study, we evaluated therapeutic effects of QLY on collagen induced arthritis (CIA) in rats and investigated its possible interactions with MTX in an integrated regimen. A computational workflow based network pharmacology study and a metabolomics analysis were carried out to further elucidate mechanisms underlying these actions.

## Materials and Methods

### Ingredients Preparation From QLY and Target Prediction

Firstly, we collected information about chemical composition of each herb in QLY formula from literatures. Those meeting certain ADME properties and drug-likeness standard (wQED > 0.3) were chosen for further target prediction analysis (Supplementary File S1) (Bickerton et al., 2012). After filtering redundant information, we obtained 234 ingredients, including 124, 47, 61, and 13 compounds from Kushen, Qingfengteng, Huangbai, and Bixie, respectively. Their structures were retrieved from the PubChem database^1^. All the chemical information was then used as data source for target prediction.

To achieve *in silico* prediction, potential targets of these ingredients were analyzed by using the drugCIPHER method, a state-of-art network-based algorithm for global prediction of compound targets (Zhao and Li, 2010). In principle, this technique predicts relationships between bioactive ingredients and candidate targets based on network-based integration of multiple pharmacological similarities by using FDA covered agents, corresponding targets and protein–protein interactions as references. In order to obtain high precision results, top 100 ranking targets in the predicted profile of each compound were kept. The reliability of prediction was further tested through the comparison of predicted targets with literature evidences. The recall efficiency was calculated by the following equation: recall = intersection of predicted targets and reported biomolecules/number of reported biomolecules × 100%.

### Enrichment Analysis and Network Construction

To identify potential pathways regulated by QLY, we carried out the enrichment analysis based on predicted targets through the fisher exact test by the aid of Gene Ontology (GO) consortium^2^ and Kyoto Encyclopedia of Genes and Genomes (KEGG) database^3^. The pathways closely related to RA were then subjected to statistical analysis, and those with *p* < 0.05 after Benjamin’s correction were deemed as significantly changed and selected for further research (Supplementary File S2). Subsequently, the results were evaluated by comparing occurrence frequency of targeted protein from prediction list of QLY to a random control represented by a Poisson binomial statistical model. With the predicted targets from selected enrichment pathways, we constructed a biomolecular network modified by QLY to elucidate its plausible anti-rheumatic mechanisms by taking protein–protein interactions and crosstalk among pathways into consideration.

### Reagents

Incomplete Freund’s adjuvant (IFA) and lyophilized immunization grade bovine type II collagen (CII) were purchased from Sigma-Aldrich (St Louis, MO, United States) and Chondex (Redmond, WA, United States), respectively. MTX tablet was the product of Sine Pharm (Shanghai, China). Methanol and acetonitrile of chromatographic grade were supplied by Merck Chemicals (Shanghai, China). 2-Chloro-L-phenylalanine was brought from Hengbai Biotechnology (Shanghai, China).

### Animals

Male SD rats (180 ± 10 g, 5–6 weeks old, supplied by Qinglongshan Laboratory Animal Company, Nanjing, Jiangsu, China) were used in this study. The animals were housed in Specific Pathogen Free (SPF) conditions to avoid any possible infections. Every four rats were kept in a separated cage. The environment was strictly controlled. The light was switched in a 12 h light on/off cycle to mimic natural rhythm. The temperature and relative humidity were set at 22 ± 2°C and 50 ± 2%, respectively. All the rats had *ad libitum* access to a standard pelleted food and boiled tap water. The animals were kept for 7 days to get accommodated to the situation prior to *in vivo* experiments. The animal experimental protocols were approved by Ethical Committee of Yijishan Hospital and strictly in accordance with the guideline for the care and use of laboratory animals (United States National Research Council, 2011).

### Induction of CIA in Rats

Induction of CIA in rats was performed according to the protocol of Chondex with minor modifications. Lyophilized CII was dissolved in 0.05 M acetic acid to obtain a solution at the concentration of 2 mg/ml, and stood overnight under 4°C. Under continuous stirring using a pestle motor (Kimble Chase, Vineland, NJ, United States), equal volume of CII solution was mixed into IFA drop-wise on the ice to obtain the stable and homogeneous emulsion. A multi-points subcutaneous injection (total volume was 0.1 ml) was carried out at day 0 with a Hamilton syringe. Seven days later, a booster injection with 0.1 ml of emulsion was administered at the base of the tail subcutaneously.

### Preparation of QLY Extraction and Chemical Composition Characterization

Prior to animal experiments, QLY decoction was prepared. Radix Sophorae Flavescentis, Caulis Sinomenium Acutum, Cortex Phellodendri Chinensis, and Rhizoma Dioscoreae Hypoglaucae were brought from Bozhou Herbal Medicine Market, and identified by Professor Jian-Wei Chen (College of Pharmacy, Nanjing University of Chinese Medicine, China). Voucher specimens (ID: QLY-2017-001-004) were deposited in the Herbarium Center, Wannan Medical College, China. The herbs were mixed at the ratio of 1.5:1.2:1:1, and extracted with boiling water for three times. Finally, the filtrates were combined and condensed to sticky extract (4 g/ml relative to crude drugs) by using a rotavapor.

A sample of the extract was diluted with methanol, filtered through a 0.45 μm filter, and then subjected to HPLC analysis to characterize the chemical composition. The detection was performed on a LC-20AT HPLC system (Shimadzu Corporation, Kyoto, Japan) coupled with a UV detector using a Thermo SCIENTIFIC C_18_ column (250 mm × 4.6 mm, 5 μm), and other analysis conditions were summarized as below. A gradient elution at flow rate of 1.0 ml/min was adopted. The mobile phase A and B were ammonium acetate (10 mM in water) and methanol-acetonitrile 1:3 (v/v) containing 10 mM ammonium acetate and 0.2% ammonia, respectively, and the elution program is shown in Supplementary File S3. The detection wavelength and column temperature were set at 215 nm and 35°C, respectively. Main signals in chromatogram were identified by the comparison with the reference compounds (magnoflorine, sinomenine, matrine, sophocarpine, and berberine, supplied by Chengdu Herbpurify Co., Ltd., Sichuan, China). Representative chromatograms and results of the quantitative analysis were also included in Supplementary File S3.

### Administration

Thirty-two CIA rats were divided into equal four groups randomly (with eight rats each), three of which were assigned as treatment groups (MTX, QLY, MTX+QLY), and the other served as model control. MTX suspended in 0.5% CMC-Na was administered to rats at the dose of 0.5 mg/kg twice a week intragastrically. QLY group was treated with QLY extract once a day at the dose of 0.3 g/kg (relative to the dry extract). MTX+QLY group received combined treatments of QLY and MTX. Eight normal animals and CIA models were lavaged with CMC-Na synchronously. Since day 1, treatments lasted for 36 days until sacrifice.

### Assessment of Arthritis

Clinical severity of arthritis in rats was assessed by two scholars in a blind manner periodically since the initial immunization. Volume of the right hind paw was quantitatively determined to evaluate the inflammation using the water displacement method. Onset and progress of arthritis were assessed by arthritis scores. Each paw was scored independently in a four grade scale (1–4), and the theoretical maximum sum was 16. The score criterion was defined as below: 1, slight swelling or redness of one toe; 2, moderate erythema and swelling; 3, severe inflammation in the entire paw; 4, swollen ankle joint and joint rigidity. To investigate radiological outcomes, digital radiography (DR) examination of limbs was carried out 3 days ahead of sacrifice on a Digital Diagnsot DR system (Philips Healthcare, Best, Netherlands).

### Sampling and Sacrifice

On day 35, 24 h urine samples of the rats were collected through automatic micturition, which was centrifuged at 12,000 rpm for 10 min. The supernate was kept at −80°C until analysis. One day later, the rats were anesthetized with chloral hydrate, and maximum amount of blood was collected via abdominal aorta into promoting coagulating and anticoagulation tubes for the serum separation and blood cell subset analysis, respectively. The serum was obtained after a centrifugation, and immediately divided into aliquots and stored at −80°C until further analyses. Thereafter, all the animals were sacrificed. Organs and hind paw were promptly dissected, and fixed in 10% buffered formalin for histological examinations.

### Hematological Analyses and Histological and Immunohistochemical Examinations

One portion of anticoagulated blood was subjected to an automated hematology system (ADVIA 120, Bayer Diagnostics, Germany) for complete blood count (CBC) analysis. Another portion was used for CD4^+^CD25^+^ T cells distribution analysis by using APC tagged CD4 and PE tagged CD25 antibodies (Multi-Sciences, Hangzhou, Zhejiang, China) on a flow cytometry (FACS Calibur system, Becton and Dickson, San Jose, CA, United States). Aliquots of serum were subjected to an AU680 biochemical analyzer (Beckman, Tokyo, Japan) for the quantitative analyses of alanine transaminase (ALT) and aspartate transaminase (AST) to evaluate possible detriments on liver. Anti cyclic citrullinated peptides antibody (anti-CCP antibody), rheumatoid factor (RF) and C-reactive protein (CRP) in serum were analyzed by using commercial available ELISA kits (Cusabio, Wuhan, Hubei, China) in accordance to the manufacturers’ protocols.

The fixed organs and limbs (decalcified in 10% EDTA for 2 weeks prior to the following experiments) were embedded in paraffin, and sectioned at 5 μm followed by staining with hematoxylin/eosin. The stained sections were observed using an Olympus BH-2 light microscope (Tokyo, Japan). Some other sections were deparaffinized and soaked with 0.3% H_2_O_2_ in 60% methanol. The specimens were then treated with citric acid (10 μM) for 1 h by the aid of microwave heating. After that, they were incubated with goat serum, specific primary and appropriate biotinylated secondary antibodies in turns. After the peroxidase staining with diaminobenzidine, signaling of proteins was visualized by the counterstaining with hematoxylin.

### Urine Sample Preparation

One hundred microliter urine was spiked into 900 μl chilled methanol–acetonitrile–water (2:2:1) solution, and another 20 μl L-2-chlorophenylalanines solution (1 mg/ml in H_2_O, served as the internal standard) was added. The mixture was vortex for 30 s, and then treated by ultrasound in an ice-water bath for 10 min. Seven hundred microliter of the supernatant was collected after a centrifugation at 13,000 rpm for 15 min under 4°C and dried using a vacuum centrifugal concentrator (Centrivap console, Labconco Company, United States). The residue was dissolved with acetonitrile–water (1:1) by the aid of ultrasonic treatment in an ice-water bath. After a high speed centrifugation, 50 μl supernatant of each sample was collected. The quality control (QC) was prepared by mixing equal amount of every sample from an identical experiment group.

### UPLC-Q-TOF/MS Analysis

UPLC-Q-TOF/MS analysis was achieved on an 1290 infinity UPLC system (Agilent Technologies, Santa Clara, CA, United States) coupled with an Triple Q-TOF 6600 mass spectrometry (AB SCIEX, Concord, ON, Canada). Analytes extracted from urine samples were separated on an ACQUITY UPLC HSS T3 column (100 mm × 2.1 mm, 1.7 μm, Waters, Milford, MA, United States) with a gradient elution program at the flow rate of 0.5 ml/min. The mobile phase was comprised of 25 mM ammonium acetate in water (phase A) and acetonitrile (phase B). The elution gradient of phase B was depicted as follows: 95% (0–0.5 min), 95% down to 40% (0.5–9 min), 40% up to 95% (9–12 min), maintained at 95% for 2 min. Optimized parameters for the mass spectrometer were as below: ion spray voltage, 5 or −4 kV (for positive and negative mode, respectively); declustering potential, 60 V; curtain gas, 25 psi; nebulizer gas of 40 psi; interface heater temperature, 650°C scan range (m/z), 50–1,200. The stability of the analysis was continuously monitored by analyzing QC samples at intervals of every four samples.

### Data Analysis

Total ion chromatograms were pre-processed with an R package, XCMS to filter redundant signals, match peaks, and accurate mass of LC-MS produced signals (Smith et al., 2006). The molecular features within deviations of 0.5 min retention time and 15 ppm mass tolerance were accepted. Those with presence less than 50% and RSD of intensity over 30% in QC samples were filtered out. The spectrometric features were then assigned by mass and retention time, and the relative intensity was normalized by Support Vector Regression method (Shen et al., 2016). Identification of metabolites were achieved by automated comparison of molecular features (retention time, mass and MS/MS spectra) to a commercial available metabolomics library (provided by Biotree Biotech, Shanghai, China, established on the same experimental platform with purified standards). A similarity threshold of 70% was used for the subsequent annotations. Obtained data were then log-transformed, mean-centered, and fed to SIMCA-P V14.1 (Umetrics, Umea, Sweden) for multivariate statistical analyses. Both unsupervised (principal component analysis, PCA) and supervised pattern discrimination (orthogonal projections to latent structures discriminant analysis, OPLS-DA) analyses were adopted to discriminate differences among groups. PCA as an initial exploratory analysis was employed to get an overview of the urine metabolites, and the further class discrimination and systematic differences extraction were achieved by the means of OPLS-DA analysis (Davis et al., 2012). Sevenfold cross validation was used to estimate the robustness and predictabilities of established model, which was further validated by 200 permutation tests. Upon the analysis, a VIP parameter was assigned to each metabolite based on the discriminatory power on models. These with VIP over 1.0 were subsequently subjected to Student’s *t*-test, and it was considered statistically significant when *P*-value < 0.05. The screened out metabolites were finally visualized as heatmaps, and mapped onto KEGG pathways. The annotated metabolites were highlighted by different colors dependent on the regulatory manners (red for up-regulation and blue for down-regulation).

The required data from pharmacological experiments were processed by using SPSS software (version 14.0, SPSS Inc., Chicago, IL, United States). Results were expressed as mean ± SEM. To evaluate differences among groups, one-way analysis of variance coupled with *post hoc* test were applied.

## Results

### Target Prediction and Network Analysis of the Anti-rheumatic Mechanisms of QLY

**FIGURE 1:**
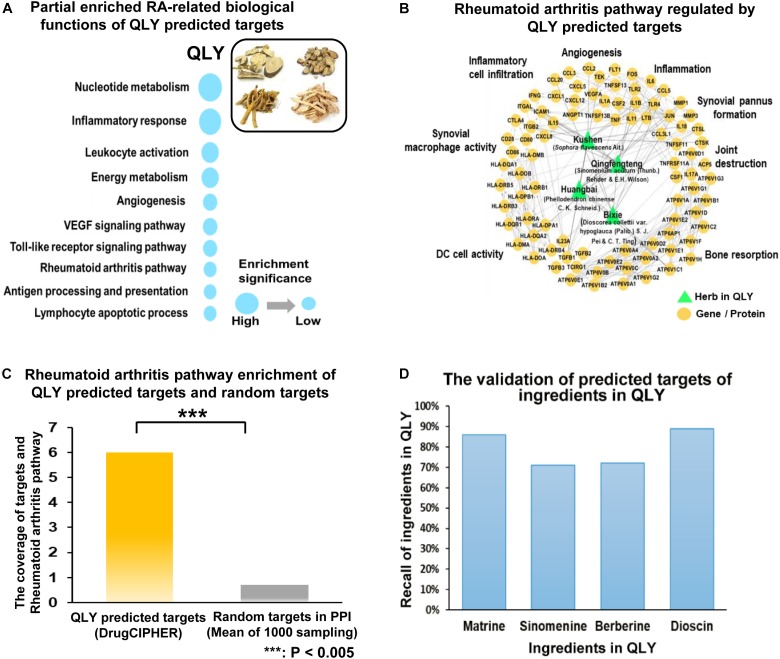
Network pharmacology analysis of anti-rheumatic mechanisms of QLY. **(A)** RA-related biological function enrichment analysis based on predicted targets (*p* < 0.05). **(B)** Comprehensive effects of QLY on rheumatoid arthritis pathway. **(C)** The statistical validation test of rheumatoid arthritis pathway enrichment analysis. **(D)** The validation of predicted targets of representative ingredients in QLY with literature evidence.

We have partially elucidated therapeutic mechanisms of QLY on RA using the network pharmacology method (Zhang et al., 2013). Considering the rapid accumulation of relevant knowledge, we updated these results in this study. To achieve a better evaluation of the clinical potentials and test the reasonability of prediction, we extracted main therapeutic targets of now available anti-rheumatic drugs from TTD^4^ and CTD^5^ database, and compared them with the predicted results. Computational analysis found predicted targets of ingredients in QLY covered most of them (Supplementary File S2). Further, potential regulated pathways implicated in pathogenesis of RA under QLY treatment were screened out from candidates based on these targets by GO and pathway enrichment analysis, and the results were ranked in Figure 1A. Apart from some well known RA related pathways involved in angiogenesis, inflammatory response, and immune reactions, the main finding was that QLY significantly altered nucleotide metabolism. Among all these targeted pathways, RA pathway is especially meaningful as it is deeply involved in the evolution of RA. Upon further examination, we found through regulation of this pathway QLY could exert effects on many pathological aspects of RA, including inflammation, joint destruction, angiogenesis and immune dysfunction (Figure 1B).

The primary statistical validation test proved that results of pathway enrichment analysis were meaningful, as we found significant statistical difference on target occurrences between QLY derived list and random control (*p* < 0.005) (Figure 1C). Further, we validated the reliability of the predicted targets based on recall of four well investigated characteristic ingredients of QLY (matrine, sinomenine, berberine, dioscin). We searched for related molecular mechanisms of the four compounds in PubMed database by the means of literature mining. The predicted targets of each ingredient are connected to the reported biomolecules in the direct or indirect manner (via protein–protein interactions or signaling pathway crosstalk). Obtained results showed that the predicted targets covered 86%, 71%, 72%, and 81% of reported biomolecular mechanisms for matrine, sinomenine, berberine, and diosgenin, respectively (Figure 1D). It suggested that predicted results were reliable.

### Effects of Treatments on Clinical Manifestations of CIA in Rats

**FIGURE 2:**
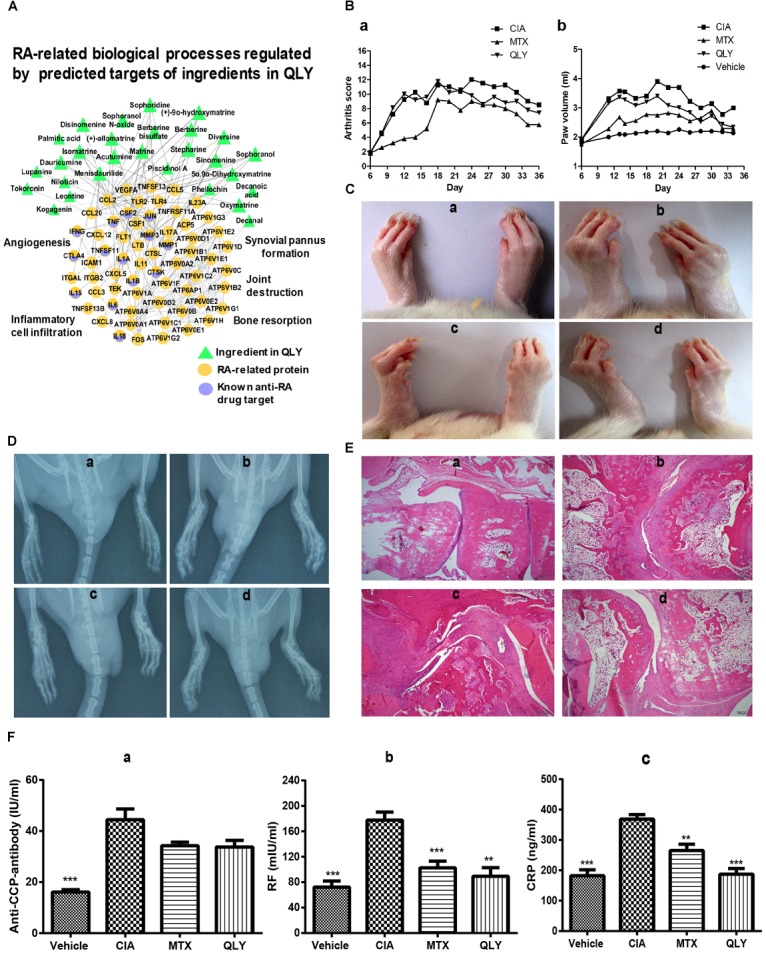
Therapeutic effects of treatments on CIA in rats. **(A)** Network pharmacological effects of ingredients from QLY on RA related biological processes. **(B)** Overall severity of arthritis in rats: a, arthritis score; b, paw edema. **(C)** Clinical manifestations of CIA; **(D)** DR examination of the hinder paw; **(E)** histological examination of ankle joints; a–d represented vehicle, CIA, MTX, and QLY, respectively. **(F)** Quantitative analyses of RA related biomarkers in serum: a–c represented anti-CCP antibody, RF and CRP, respectively; statistical significance: ^∗∗^*p* < 0.01 and ^∗∗∗^*p* < 0.001 compared with MTX group.

According to the target prediction and biological function enrichment analysis, QLY treatment could inhibit angiogenesis, inflammatory infiltration, bone resorption, and pannus formation, and subsequently protect joints from destruction and alleviated severity of arthritis (Figure 2A). Evidences from *in vivo* experiment solidly supported these claims.

Twelve days after the first induction, significant edema was developed in paws of rats, and synchronously, arthritis score was remarkably increased (Figure 2B). Since day 25, local inflammation was gradually eased, but significant deformation of joints and limitation of motion occurred in CIA rats. MTX exhibited efficient therapeutic effects on CIA, indicated by eased inflammation and reduced arthritis score throughout the experimental period. CBC analysis found some additional clues to validate its anti-inflammatory effects. Levels of both white blood cell and lymphocyte increased under CIA conditions, and MTX brought all these parameters down (data not provided). Similar but weaker effects of QLY were found (Figure 2C). Such conclusion was also obtained from DR examination. Compared with normal animals, joint structures of CIA rats were extensively damaged. It found vague and narrowed joint spaces, together with obvious cartilage ossification and bone loss. The rats received MTX/QLY treatments exhibited much clearer joints space, and joint cavity fusion and bone resorption were also suppressed (Figure 2D). These evidences suggested QLY possessed substantial therapeutic effects on CIA in rats.

### Effects of Treatments on Pathological Changes in CIA Rats

Histological examination found totally intact joints structures in normal animals, but severe damages on the joints of CIA rats, including fibrous hyperplasia, inflammatory cells infiltration, cartilage loss, and bone erosion. No obvious histological improvements were achieved under MTX treatments, while QLY alleviated these pathological conditions a lot. The joints cavity narrowing, synovial hyperplasia, bone degradation and cartilage damages were all efficiently inhibited (Figure 2E). By the comparison, obvious advantages of QLY were revealed.

To further understand therapeutic mechanisms of the treatments on CIA and predict their effects on the prognosis of disease, we analyzed levels of some RA related biomarkers in serum. Overall, all the investigated parameters were significantly elevated in CIA rats, but restored by the treatments. Upon the comparison, we found QLY performed better than MTX. Unlike RF and CRP, level of anti-CCP antibody was not so sensitive to these treatments (Figure 2F).

Collective results suggested QLY could possess an immunomodulation effect *in vivo*. Further investigations found it had little influence on the histological structures of immune organs (Supplementary File S4), but affected the distribution and differentiation of T cells in both spleen and thymus. Generally, both MTX and QLY decreased the population of CD4^+^ cells in CIA rats, and slightly reduced the production of IFN-γ (Supplementary File S5). As RA is believed as a Th1 cells driven immune disease, these changes are favorable to the improvement of pathological conditions. The expression of FOXP3 was down-regulated under CIA conditions, and both the treatments achieved no effect on it. The flow cytometric analysis found reduced population of CD4^+^CD25^+^ cells in the peripheral blood, which was even aggravated under MTX treatment. Although QLY could not raise this level, the combination treatment exhibited a tendency of recovery (Supplementary File S6). These clues hinted that QLY could restore the immune homeostasis in CIA rats mainly via down-regulation of Th1 cells.

### QLY Alleviate the Toxicity of MTX on Rats

**FIGURE 3:**
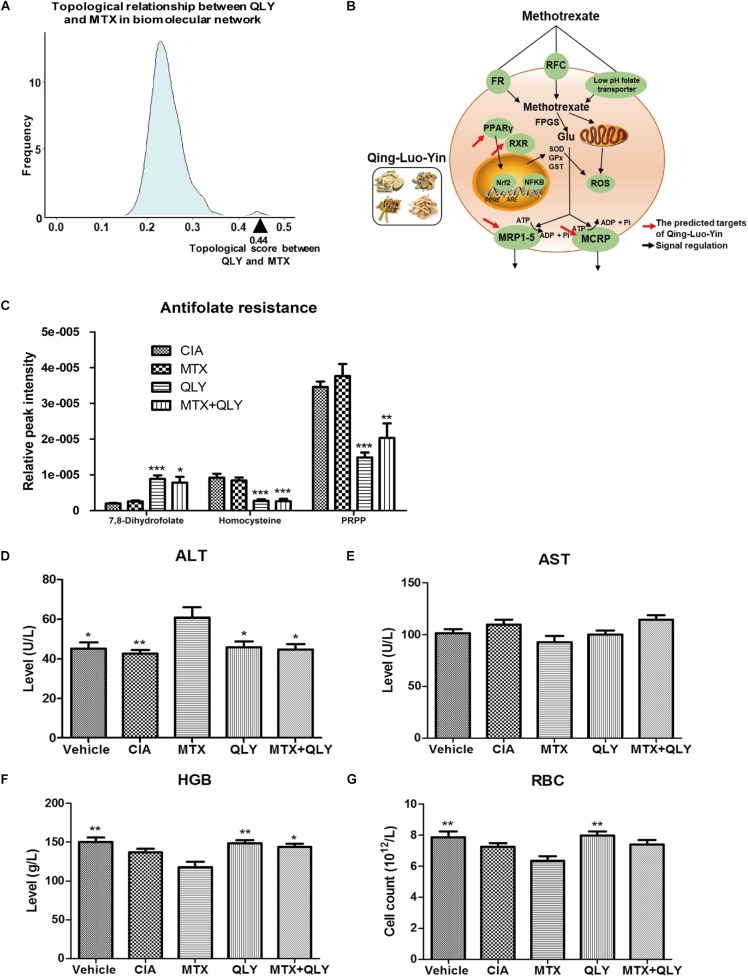
QLY alleviated MTX induced side effects in rats. **(A)** Topological relationship between QLY and MTX in the RA-related biomolecular network modeling. **(B)** Possible mechanism underlying antagonistic effects of QLY against MTX induced toxicity revealed by network pharmacological analysis. **(C)** Effects of QLY on levels of intermediates in folate metabolism. **(D,E)** Levels of ALT and AST in serum. **(F,G)** Levels of HGB and RBC in the whole blood. Statistical significance: ^∗∗∗^*p* < 0.05 and ^∗∗^*p* < 0.01 compared with MTX group.

As the network target method is a powerful tool to predict possible interactions in a treatment involves multiple bioactive components for its latent network topology properties (Li et al., 2011), we used this technique to assess theoretical feasibility of the combined regimen with MTX and QLY. As shown in Figure 3A, the predicted targets of QLY are adjacent to those of MTX, which hinted that QLY and MTX might produce combined effects. Further, the network analysis found that QLY can partially offset toxicity related signaling changes induced by MTX, which are mainly related to oxidative stress response and energy metabolism (Figure 3B). Metabolomics analysis found that QLY induced significant antifolate resistance *in vivo*. Because folate depletion is main factor leading to toxicity during a long term MTX treatment, the effects of QLY on folate metabolism are meaningful. Contrary to MTX, QLY significantly increased levels of 7,8-dihydrofolate, homocysteine and 5-phosphoribosyl 1-pyrophosphate, and exhibited antagonistic effects against MTX concerning folate metabolism in the combined treatment (Figure 3C).

The *in vivo* experiment provided more direct evidences to support the theory above. After repeated MTX administration, some rats exhibited gastrointestinal reactions, including nausea, diarrhea and dyspepsia. In the late stage of the treatment, most rats exhibited signs of anemia, such as reduced activity, pale complexion and mental fatigue, but no abnormal reactions were found in MTX+QLY group. Although histological examination found no obvious damages of liver (Supplementary File S4), level of ALT in serum was significantly elevated under MTX treatment, while no obvious change happened to AST (Figures 3D,E). Meanwhile, MTX induced decrease of hemoglobin (HGB) and red blood cell (RBC) in rats, and the combined use of QLY partly restored the abnormal changes (Figures 3F,G). Similar antagonistic effects of MTX and QLY on platelet were observed. These clues confirmed the hepatic and hematological toxicities of MTX (Kivity et al., 2014), and hinted QLY possessed protective effects on MTX induced hepatic injury and myelosuppression.

### Treatments Changed Metabolic Patterns of CIA Rats

**FIGURE 4:**
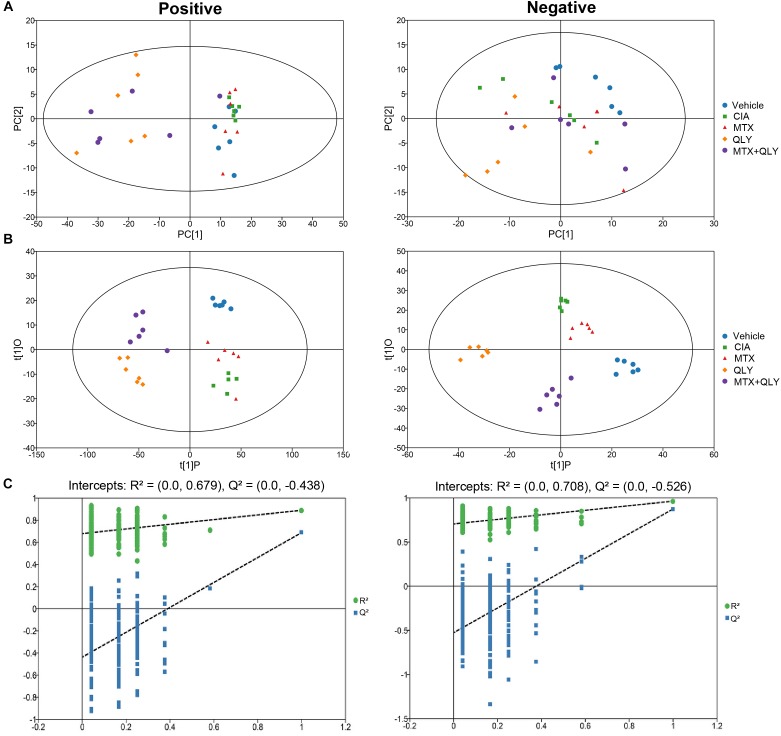
Multivariate statistical analyses of metabolic characters of urine samples acquired by UPLC-Q-TOF/MS. **(A)** Scores plot of PCA. **(B)** Scores plot of OPLS-DA. **(C)** Results of cross validation.

To maximate the information load, fingerprints of urine samples were acquired in both negative and positive modes. UPLC-Q-TOF/MS analysis detected a total of 5,949 and 60,930 signals under the negative and positive modes, respectively (Supplementary Files S7, S8). Among them, 190 and 633 metabolites were identified by using the commercial available metabolic library (Supplementary Files S9, S10). Based on global features of the raw data, we carried out PCA and OPLS-DA analyses to differentiate groups and visualize the metabolic differences among groups. PCA analysis exhibited a separation tendency of groups, but the results were not satisfying (Figure 4A). As a supervised learning method, OPLS-DA filters out non-essential variations, and notably improves the accuracy of classification. In this study, score plots from OPLS-DA models clearly discriminated all the groups under both negative and positive modes. It was revealed that the endogenous substance metabolisms of CIA rats were obviously disrupted. Upon QLY and MTX treatments, the metabolic profile of CIA rats was altered. Score plots of the two groups situated in the distinct positions in the map, which hinted different mechanisms were involved in their therapeutic actions on CIA (Figure 4B). Results from cross validation suggested the model was robust and had good predictabilities (Figure 4C).

**FIGURE 5:**
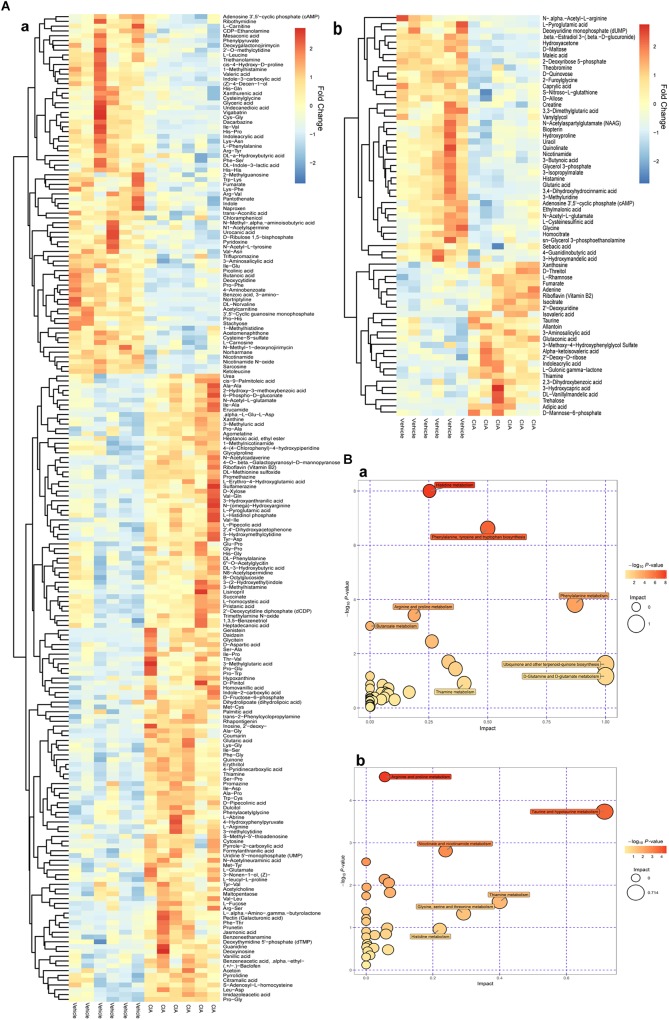
Altered metabolic profile of CIA rats. **(A)** Discriminating metabolites differentiated CIA and normal rats (VIP > 1, *p* < 0.05); **(B)** Metabolic pathways involved in the pathogenesis of CIA; a, positive mode, b, negative mode.

By comparison between normal and CIA rats, potential biomarkers involved in pathogenesis of CIA were screened out based on OPLS-DA analysis with the predetermined rules (VIP > 1; *p* < 0.05) (Figure 5A). The discriminatory metabolites were then annotated by KEGG database. It was found the perturbed pathways mainly covered amino acid and fatty acid metabolisms (Figure 5B). Generally, short and medium chain fatty acids such as butanoic acid, valeric acid, undecanedioic acid and caprylic acid were decreased but the long chain ones including *cis*-9-palmitoleic acid, palmitic acid and heptadecanoic acid were increased. The regulation on amino acid metabolism was diverged and sophisticated. Levels of arginine, aspartic acid, *N*-(omega)-hydroxyarginine and glutamate were raised, while concentrations of norvaline, phenylalanine, sarcosine and leucine were reduced. Energy metabolism in CIA rat was boosted, which was suggested by high amount of intermediates from tricarboxylic acid (TCA) cycle (fumarate, succinate, and isocitrate) in the urine. Besides, we found increased 3-hydroxybutyric acid and creatine, and disordered vitamin B profile (high riboflavin and thiamine but low nicotinamide) under CIA conditions (Table 1). These evidences further validated the disruption of energy metabolism in CIA rats. MTX restored most of the abnormal metabolic changes, while its effect on amino acid metabolism was weak. The selective restoration of MTX on the disordered metabolic state of CIA rats changed the position of score plots in OPLS-DA diagram (situated between CIA and normal rats). This phenomenon indicated the substantial recovery of CIA rats under MTX treatment. Different from the selective regulation of MTX, QLY treatment universally compromised all of the endogenous substances metabolisms (Table 1), which gave the treated rats a totally different metabolic profile from the others (Figure 4).

**Table 1:** Regulatory effects of treatments on the metabolic profile of CIA rats.

	Vehicle	CIA	MTX	QLY	MTX+QLY
Arginine	7.62 × 10^−4^	1.23 × 10^−3^	1.17 × 10^−3^	3.87 × 10^−4^	4.53 × 10^−4^
Aspartic acid	4.51 × 10^−5^	5.25 × 10^−5^	4.85 × 10^−5^	1.74 × 10^−5^	2.27 × 10^−5^
*N*-(omega)-Hydroxyarginine	1.64 × 10^−5^	3.60 × 10^−5^	3.85 × 10^−5^	9.83 × 10^−5^	1.02 × 10^−5^
Glutamate	1.71 × 10^−5^	2.09 × 10^−5^	2.09 × 10^−5^	7.95 × 10^−6^	9.37 × 10^−6^
Norvaline	4.23 × 10^−4^	8.73 × 10^−5^	1.01 × 10^−4^	7.53 × 10^−5^	8.14 × 10^−5^
Phenylalanine	3.05 × 10^−4^	1.98 × 10^−4^	2.75 × 10^−4^	5.02 × 10^−5^	8.61 × 10^−5^
Sarcosine	2.22 × 10^−4^	1.08 × 10^−4^	1.85 × 10^−4^	7.24 × 10^−5^	1.49 × 10^−4^
Leucine	7.96 × 10^−5^	5.36 × 10^−5^	5.00 × 10^−5^	1.47 × 10^−5^	1.84 × 10^−5^
Urea	2.04 × 10^−3^	2.34 × 10^−3^	2.33 × 10^−3^	6.32 × 10^−4^	8.05 × 10^−4^
*cis*-9-Palmitoleic acid	7.75 × 10^−6^	1.21 × 10^−5^	1.03 × 10^−5^	5.60 × 10^−6^	4.31 × 10^−6^
Palmitic acid	3.78 × 10^−3^	6.29 × 10^−3^	3.73 × 10^−3^	1.01 × 10^−3^	1.03 × 10^−3^
Heptadecanoic acid	6.06 × 10^−5^	1.16 × 10^−4^	2.54 × 10^−5^	8.78 × 10^−6^	8.22 × 10^−6^
Butanoic acid	7.67 × 10^−5^	1.37 × 10^−5^	6.60 × 10^−5^	8.89 × 10^−6^	4.45 × 10^−5^
Valeric acid	2.10 × 10^−5^	8.93 × 10^−6^	1.39 × 10^−5^	3.43 × 10^−6^	3.69 × 10^−6^
Undecanedioic acid	8.83 × 10^−5^	5.59 × 10^−5^	5.72 × 10^−5^	1.57 × 10^−5^	4.64 × 10^−5^
Caprylic acid	1.38 × 10^−4^	7.85 × 10^−5^	1.12 × 10^−4^	1.09 × 10^−4^	1.01 × 10^−4^
Carnitine	9.51 × 10^−5^	5.53 × 10^−5^	8.22 × 10^−5^	3.72 × 10^−5^	3.06 × 10^−5^
Ethylmalonic acid	6.34 × 10^−5^	2.90 × 10^−5^	5.75 × 10^−5^	3.29 × 10^−5^	6.10 × 10^−5^
Creatine	2.95 × 10^−3^	3.91 × 10^−3^	5.80 × 10^−3^	4.06 × 10^−3^	4.89 × 10^−3^
3-Hydroxybutyric acid	4.79 × 10^−5^	5.29 × 10^−5^	5.18 × 10^−5^	2.25 × 10^−5^	2.26 × 10^−5^
Fumarate	4.84 × 10^−6^	7.02 × 10^−6^	1.10 × 10^−5^	5.94 × 10^−6^	7.36 × 10^−6^
Succinate	1.41 × 10^−5^	1.95 × 10^−5^	1.76 × 10^−5^	1.2 × 10^−5^	9.54 × 10^−6^
Isocitrate	1.95 × 10^−4^	3.80 × 10^−4^	2.72 × 10^−4^	1.26 × 10^−4^	1.10 × 10^−4^
Maleic acid	1.82 × 10^−3^	7.99 × 10^−4^	2.04 × 10^−3^	1.35 × 10^−3^	2.18 × 10^−3^
Nicotinamide	9.64 × 10^−3^	3.88 × 10^−4^	5.78 × 10^−3^	1.86 × 10^−3^	2.42 × 10^−3^
Riboflavin	6.04 × 10^−6^	1.37 × 10^−5^	1.50 × 10^−5^	5.29 × 10^−6^	3.74 × 10^−6^
Thiamine	4.53 × 10^−3^	1.29 × 10^−2^	7.97 × 10^−3^	3.97 × 10^−4^	2.95 × 10^−4^

*All these metabolic intermediates were detected by UPLC-Q-TOF/MS method, and the relative levels in urine were evaluated based on their contribution to the sum of signal intensities.*

### Metabolic Changes Involved in Therapeutic Actions of QLY on CIA

**FIGURE 6:**
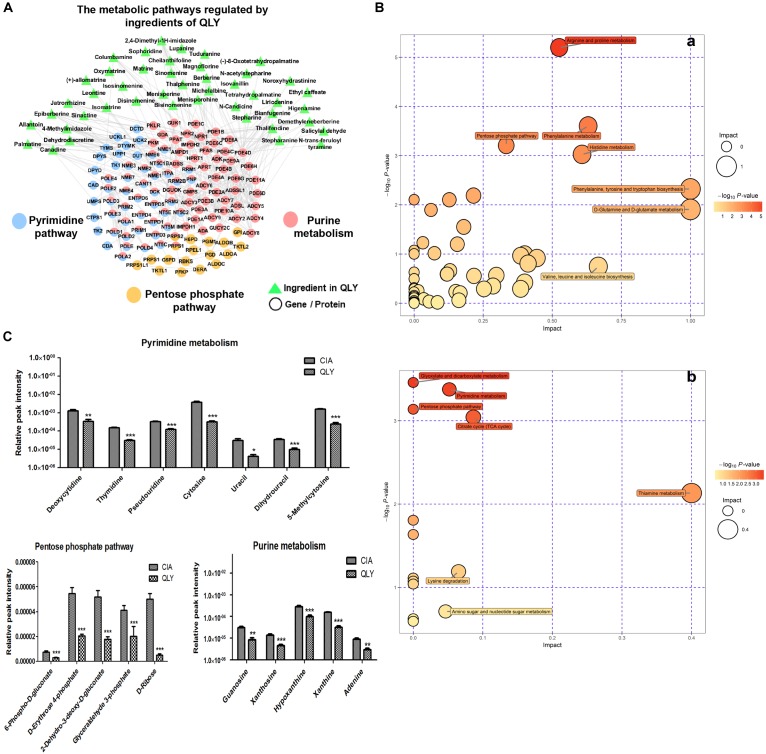
Regulatory effects of QLY on nucleotide metabolism. **(A)** Possible effects of QLY on nucleotide biosynthesis related metabolic pathways predicted by network pharmacological method. **(B)** Perturbed metabolic pathways in CIA rats under QLY treatment: a, positive; b, negative. **(C)** Effects of QLY on levels of intermediates in nucleotide biosynthesis related pathway: a, pyrimidine metabolism, b, pentose phosphate pathway, c, purine metabolism.

The biological function enrichment analysis and metabolomics study found QLY could intervene in the nucleotide metabolism (Figure 1A). To fully interpret its clinical implication and get a better understanding of the anti-rheumatic metabolism of QLY, we dug deeply into relevant data, and found pyrimidine metabolism, purine metabolism and pentose phosphate pathway (PPP) could all be altered by QLY (Figure 6A). The computational result suggested chemical ingredients of QLY can simultaneously target multiple genes/proteins involved in nucleotide metabolism, and suppress some abnormal cellular functions fueled by the high metabolism status.

Subsequently, we then test this hypothesis with metabolomics evidences. OPLS-DA analysis revealed 39 and 413 discriminatory metabolites between CIA and QLY treated rats at negative and positive mode, respectively (Supplementary Files S11, S12). Besides from previous mentioned amino acid, energy and thiamine metabolisms, we noticed nucleotide biosynthesis relevant metabolic pathways were also affected a lot by QLY (Figure 6B). Enriched KEGG pathway analysis demonstrated that PPP and pyrimidine metabolism in CIA rats were significantly down-regulated under QLY treatment, which contributed to the discrimination of CIA and QLY groups. Relative quantitative analysis demonstrated levels of both free pyrimidine bases (cytosine, uracil, dihydrouracil and 5-methylcytosine) and pyrimidine nucleosides (deoxycytidine, thymidine, and pseudouridine) were remarkably reduced by QLY compared with CIA models. Similar inhibitory effects on PPP were also observed. Many intermediates in this metabolic pathway including 6-phospho-D-gluconate, D-erythrose-4-phosphate, 2-dehydro-3-deoxy-D-gluconate, glyceraldehyde 3-phosphate and D-ribose were all down-regulated. By further manual comparison, we found purine metabolism was suppressed by QLY too. Some important endogenous substances implicated in purine metabolic pathway including hypoxanthine, xanthine, adenine, xanthosine, and guanosine were all reduced (Figure 6C). These evidences showed QLY compromised nucleotides biosynthesis and disrupted nucleic acid metabolism. For the important role of nucleotides in cell division, these effects could have a close relationship with the protective effects on joints in CIA rats via inhibition of synovial hyperplasia, and also could influence the immune functions *in vivo*.

## Discussion

By accumulation of knowledge into the pathogenesis, rheumatologists designed many novel therapeutic approaches. However, even the most promising biological therapies cannot obtain sustained remission of RA in the long term. While under the guidance of holistic strategy, TCM formulas can usually substantially alleviate severity of RA by controlling systemic symptoms. QLY has been extensively used in clinical practice for several years (Zhang, 2015; Fan and Li, 2016). Previous studies have found that QLY efficiently inhibited angiogenesis in rheumatoid synovium (Li et al., 2003; Liu, 2012). As well accepted, fibroblast-like synoviocytes in RA (RA-FLS) play a key role in degradation of joint structure and provoking chronic inflammation (Bartok and Firestein, 2010). From this point of view, inhibition on synovial angiogenesis will contribute a lot to the protection of QLY on joints by blocking nutrition supply to the synovial hyperplasia. Despite of these findings, mechanism underlying therapeutic actions of QLY on RA is far away from being well elucidated. For example, clinical observations found obvious improvements of immune mediated symptoms, and down-regulation of RA related biomarkers under QLY treatment, while no reasonable theory has been deduced for this so far (Zhang, 2015; Fan and Li, 2016). By using an integrative platform of TCM network pharmacology, we uncovered some potential target network of QLY against RA-related key processes in a previous report (Zhang et al., 2013). It evidenced the overall mechanism of QLY on RA for the first time, however, as a theory deduced from mathematical models, these discoveries should be further validated by experimental evidences. In the present study, we updated relevant results and paid more attentions on RA related metabolic changes using a integrating strategy coupled with network pharmacology and metabolomics methods.

The *in vivo* experiments provided sufficient evidences to support the therapeutic effects of QLY on CIA (Figure 2). Integrating network pharmacology and metabolomics study did not only further confirm its anti-rheumatic potential but also aid a better understanding of the underlying mechanisms from a global protective. Of note, this study revealed a novel metabolism mediated therapeutic mechanism of QLY. The most discriminating altered metabolites from both RA patients and animal models included amino acids, lipids, ketone bodies and TCA cycle related intermediates (Castro-Santos et al., 2015; Guma et al., 2016). It reflected the accelerated degradation of tissues and increased energy consumption. A cohort analysis found higher basal metabolic rate of RA patients than that of health population, and the extra energy consumption was believed to be in close relationship with the highly energy dependent inflammatory responses (Metsios et al., 2008). From this point of view, inhibition of energy metabolism is beneficial to the amelioration of RA. Network pharmacological analysis suggested QLY could intervene into the energy metabolism (Figure 1A) via a possible means of regulation on fatty acid oxidation indicated by its effects on PPARγ (Figure 3B). This hypothesis was solidly validated by the metabolomics evidences. We found increased long chain fatty acids in urine, which indicated enhanced lipid mobilization. Meanwhile, levels of small molecular fatty acids were decreased. It can be concluded that, under CIA conditions glycometabolism was insufficient to meet the energy demands, and fatty acids were utilized as an important alternative energy sources. Enhanced catabolism of fatty acids produced more ketone bodies, and the presence of 3-hydroxybutyrate in urine reflected respiratory chain deficiency and increased oxidative stress *in vivo* (Sato et al., 1992). Carnitine is an intermediate associated with β-oxidation of fatty acids. Reduced carnitine coupled with increased 3-hydroxybutyrate indicated altered lipid catabolism and reduced antioxidant capacity (Sato et al., 1992). QLY restored all these abnormal changes (Supplementary Files S11, S12). It significantly brought the basal metabolic rate down and altered vitamin B family profile in CIA rats (Table 1). The compromise of fatty acids and carbohydrate metabolisms could serve as an important indicator for the amelioration of CIA, and raised level of nicotinamide hinted functional restoration of respiratory chain. The increase of energy utilization subsequently resulted in reduced generation of ROS, and improved inflammatory microenvironment eventually. According to TCM theory, QLY is characterized as a Cold natured formula, and used to expel “Pathogenetic Hot” in Hot Syndrome-related RA patients. This characteristic indicates its potent negative effects on energy metabolism, and the clinical implications. The present integrating mechanism study provided direct clues to validate this theory and clarify its nature.

The network pharmacology analysis suggested the therapeutic efficacy of QLY on CIA were the sum of multi-target effects from the bioactive ingredients. Among the targeted pathways and biological processes, many of them are deeply implicated in evolution of RA. By inhibiting inflammatory response, angiogenesis, and VEGF signaling pathway, QLY can alleviate the severity of both experimental arthritis and RA. Meanwhile, it exhibited potent effects on immune functions (Figure 1A). It could contribute even more to the alleviation of RA, as the pathological changes in RA are mainly caused by immune dysfunction. These results shed some lights on elucidation of anti-rheumatic mechanisms of QLY, but it also raises a fundamental question: where does its immunoregulatory activity come from? We noticed that the regulated pathway ranked in the first position by QLY was nucleotide metabolism. Further analysis found QLY simultaneously altered pyrimidine, purine, and pentose phosphate metabolisms (Figure 6A). It suggested nucleotide biosynthesis related pathways could be closely connected to the anti-rheumatic potential of QLY, and also important to resolve the question above. PPP is a fundamental component of cellular metabolism. It provides precursors for nucleotide, which is essential to support the high proliferation rate of cells under some pathological conditions. Besides, it defeats oxidative stress by generation of NADPH, and balances redox homeostasis in cells (Stincone et al., 2015). Because of these reasons, PPP could be a potential therapeutic target for cancers (Yu et al., 2015). Similarly, via inhibition of PPP, QLY could suppress the hyperproliferation of RA-FLS, and elicit DNA damage response by accumulation of intracellular ROS. Besides, PPP is implicated in inflammatory reactions, and the modification on it could be beneficial to the alleviation of systematic symptoms in CIA rats by controlling critical molecular events involved in immune responses (Nagy and Haschemi, 2015; Ham et al., 2016). Hence, inhibition on PPP by QLY is essential to the alleviation of CIA, and similarly, the regulation on pyrimidine and purine metabolisms would also yield some profound benefits (Figure 6C). As rapid expansion of autoreactive lymphocytes initiates pathological changes of RA, inhibition on them is a reasonable therapeutic strategy. Agents targeting pyrimidine metabolism will block their excessive proliferation, and are usually used as immunosuppressant for treatments of immune diseases (Peres et al., 2017). As the best known representative, leflunomide has been successfully applied in the therapy of RA for decades. Treatment with QLY significantly suppressed pyrimidine metabolism, which could contribute to the immunosuppressive effects (suppression on the clone and differentiation of Th1 cells) and down-regulation of RA-related biomarkers in CIA rats. Altered purine metabolism could be involved in the therapeutic actions of QLY on CIA too. By depleting the pool of purine required for DNA synthesis, QLY could exert a cytostatic effect on T lymphocytes (Figure 1A), and exhibit an immunosuppressive activity *in vivo* (Allison and Eugui, 1996). But if suppression on purine metabolism is beneficial to RA patients is still elusive, for adenosine derivatives usually elicit anti-inflammatory immune responses (Cekic and Linden, 2016).

The computational prediction suggested different mechanisms were involved in the anti-rheumatic effects of MTX and QLY (Figure 3A).Perhaps due to the poor optimization, we didn’t notice significant synergetic efficacy of the two treatments on CIA in this study. But QLY notably reduced MTX induced side effects. The collective clues demonstrated QLY elicited antifolate resistance was the main factor leading to this phenomenon. In the combination treatment, QLY significantly offset the negative effects of MTX on folate metabolism, and greatly restored levels of some important intermediates (Figure 3C). This finding preliminarily proved the rationality of the combined regimen, as it would greatly improve MTX tolerance in patients. However, we should also take some negative possibilities into consideration, since inhibition of folate metabolism is one of the fundamental mechanisms of MTX in treatment of RA. Also, QLY induced antifolate resistance would affect the adenosine metabolism, which has been revealed in this study (Supplementary File S13). This change could exert pronounced effects in the combination regimen, as regulation on levels of adenosine and its derivatives is believed deeply involved in anti-inflammatory effects of low dose MTX treatment on RA. Therefore, more researches are needed to fully evaluate the feasibility of the combination treatment.

According to prescription principles of TCM, the components in a formula should be defined as four types based on their functions, that is, Monarch, Minister, Assistant and Guide. The main characteristic of QLY is that large amount of Kushen is applied as monarch drug, because this herb is usually used for external purposes. Qingfengteng and Huangbai are deemed as minister drugs, while Bixie just functions as the Assistant/Guide. Hence, although all these herbals contribute to the therapeutic effects of QLY on RA, the importance varies a lot. By examination of Figure 1B, we found some evidences to firmly support the composition principle. The hierarchy of Kushen, Qingfengteng, and Huangbai in the formula were highlighted by lots of converges with RA-related signals, but Bixie seems having less influence on the anti-rheumatic potentials. Under the guide of serum pharmacochemistry, a previous report found main bioactive ingredients in QLY could be matrine, berberine, sinomenine, and their derivatives, as they are main chemical ingredients from major components in QLY, and can enter the circulation directly after oral administration (Yang, 2010). We further proved this conclusion. All of the three compounds exhibit potent effects on RA-related processes (Figure 2A). Among them, the therapeutic effects of sinomenine on RA have been well validated (Xu et al., 2008). Also, there are plenty of reports concerning the anti-rheumatic potentials of berberine. Available evidences suggested berberine can protect joints by inhibiting RA-FLS, and alleviate systemic symptoms by suppressing hyper-activated immune system (Wang et al., 2011; Wang X. et al., 2017). By contrast, there are few reports about anti-rheumatic effects of matrine, and we think its potentials are greatly underestimated. Wang S. J. et al., 2017) found matrine and its derivatives could efficiently intervene into the energy metabolism, which could be associated with the Cold nature of QLY, and its clinical application. Subsequently, we will further investigate the anti-RA related bioactivities of matrine, and evaluate its contributions to the formula.

## Data Availability Statement

All relevant data about the findings are included in this manuscript and Supplementary Files.

## Author Contributions

SL conceived the idea of the study. SL and YLi supervised the study. XW and SL performed the computational analysis. JZ and YLiu performed the *in vivo* experiments and collected the pharmacological data. JY and QL identified the chemical composition of QLY. JZ performed the metabolomics study. All authors participated in the interpretation of experimental results and drafting the manuscript.

## Conflict of Interest Statement

The authors declare that the research was conducted in the absence of any commercial or financial relationships that could be construed as a potential conflict of interest. The handling Editor and reviewer S-BS declared their involvement as co-editors in the Research Topic, and confirm the absence of any other collaboration.

## Footnotes

1 https://pubchem.ncbi.nlm.nih.gov/

2 http://www.geneontology.org

3 https://www.kegg.jp

4 http://bidd.nus.edu.sg/group/cjttd/

5 http://ctdbase.org/

## Supplementary Material

FILE S1 Ingredient information of QLY.

FILE S2 Biological function enrichment analysis of known anti-RA targets and predicted targets of QLY.

FILE S3 HPLC-UV chromatograms and quantitative results of QLY chemical composition.

FILE S4 Histological examinations of main organs of rats.

FILE S5 Immunohistochemical examinations of spleen and thymus of rats.

FILE S6 Distribution of CD4^+^CD25^+^ cells in peripheral blood of rats.

FILE S7 Raw spectrometric data under negative mode.

FILE S8 Raw spectrometric data under positive mode.

FILE S9 Identified metabolites under negative mode.

FILE S10 Identified metabolites under positive mode.

FILE S11 Discriminating metabolites among groups under negative mode.

FILE S12 Discriminating metabolites among groups under positive mode.

FILE S13 Effects of QLY on adenosine metabolism in CIA rats.
