# Supplementary material for: Integrating Network Pharmacology and Metabolomics Study on Anti-rheumatic Mechanisms and Antagonistic Effects Against Methotrexate-Induced Toxicity of Qing-Luo-Yin
Source: Front Pharmacol. 2018 Dec 18;9:1472. doi: 10.3389/fphar.2018.01472 (PMC6305420; doi:10.3389/fphar.2018.01472)
Supplement: FILE S1 — Ingredient information of QLY. [file Data_Sheet_1.ZIP › Supplementary/S3 HPLC-UV chromatograms and quantitative results of QLY chemical composition.docx]

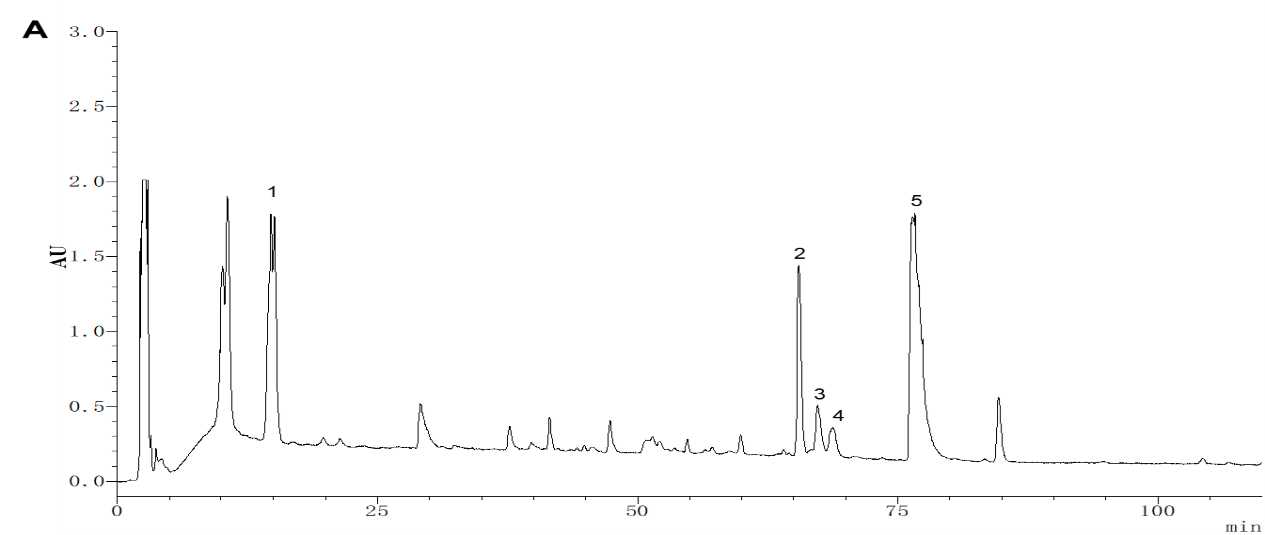


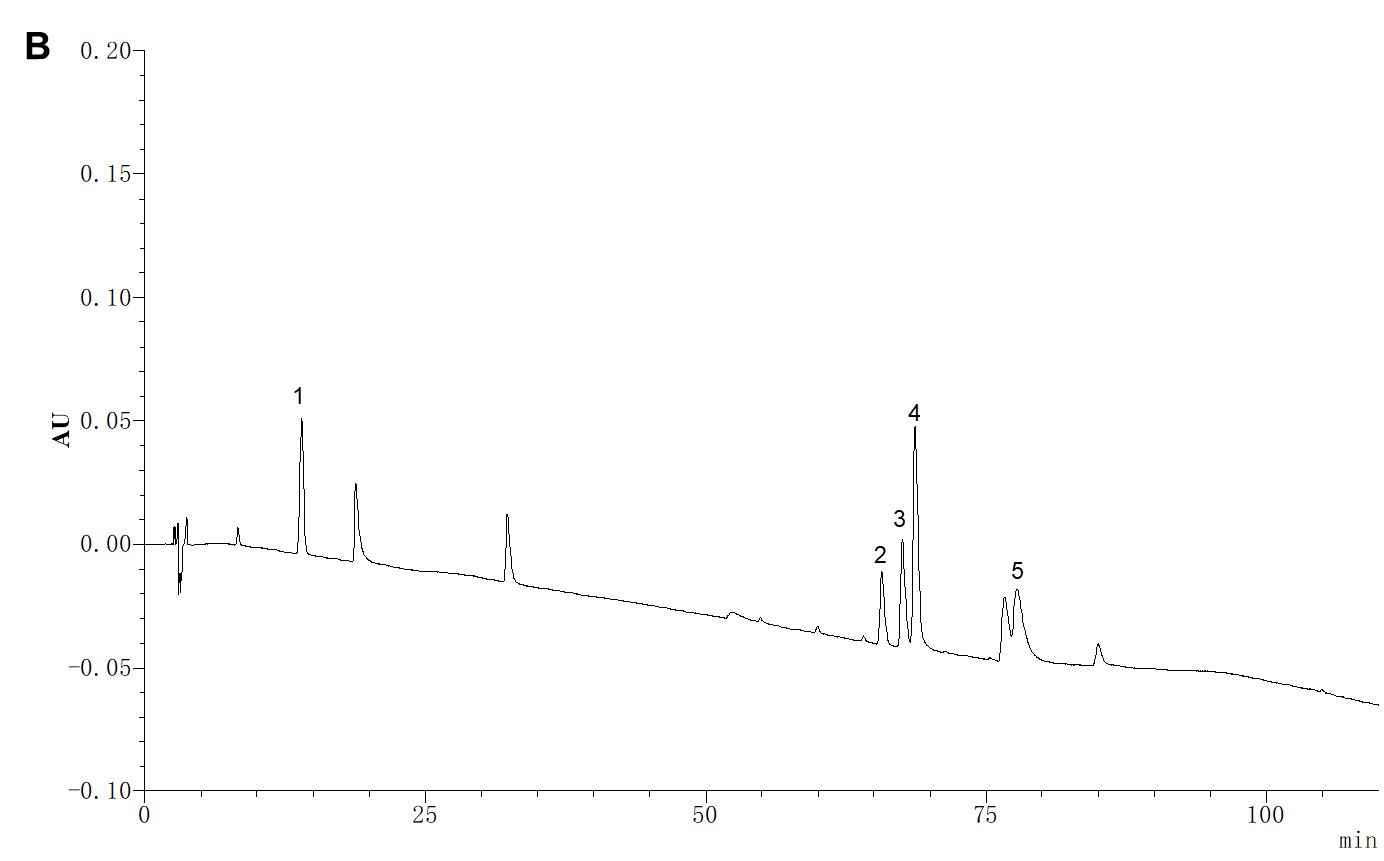


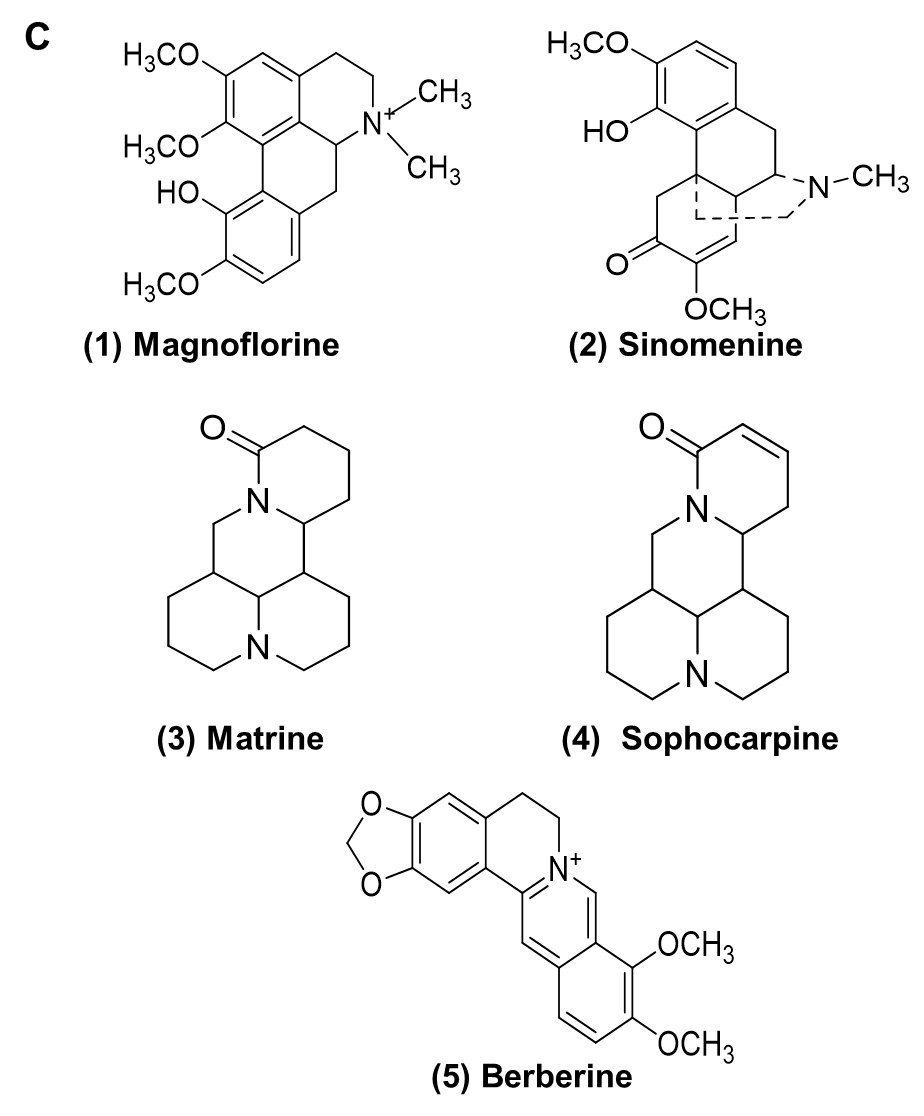


**Figure S3.** HPLC-UV chromatograms of (A) Qing-Luo-Yin and (B) 5 mixed bioactive markers with the detection at 215 nm: (1) Magnoflorine; (2)Sinomenine; (3)Matrine; (4)Sophocarpine; (5) Berberine. (C) Chemical structures of the major constituents identified from Qing-Luo-Yin.

| **Table S3-1 Solvent Gradient Program of HPLC Analysis** | | |
| --- | --- | --- |
| Time (min) | A (%) | B (%) |
| 0 | 95 | 5 |
| 35 | 83 | 17 |
| 60 | 73 | 27 |
| 70 | 70 | 30 |
| 90 | 68 | 32 |
| 105 | 60 | 40 |
| 110 | 60 | 40 |

**Table S3-2 The quantitative results of QLY major constituents**

| Sample solution | Extract |
| --- | --- |
| Compounds | **Contents of Compounds (mg/g)** |
| Magnoflorine | 24.04 |
| Sinomenine | 30.89 |
| Matrine | 10.22 |
| Sophocarpine | 16.16 |
| Berberine | 61.00 |
