# Supplementary figures and images for: Integrating Network Pharmacology and Metabolomics Study on Anti-rheumatic Mechanisms and Antagonistic Effects Against Methotrexate-Induced Toxicity of Qing-Luo-Yin
Source: Front Pharmacol. 2018 Dec 18;9:1472. doi: 10.3389/fphar.2018.01472 (PMC6305420; doi:10.3389/fphar.2018.01472)

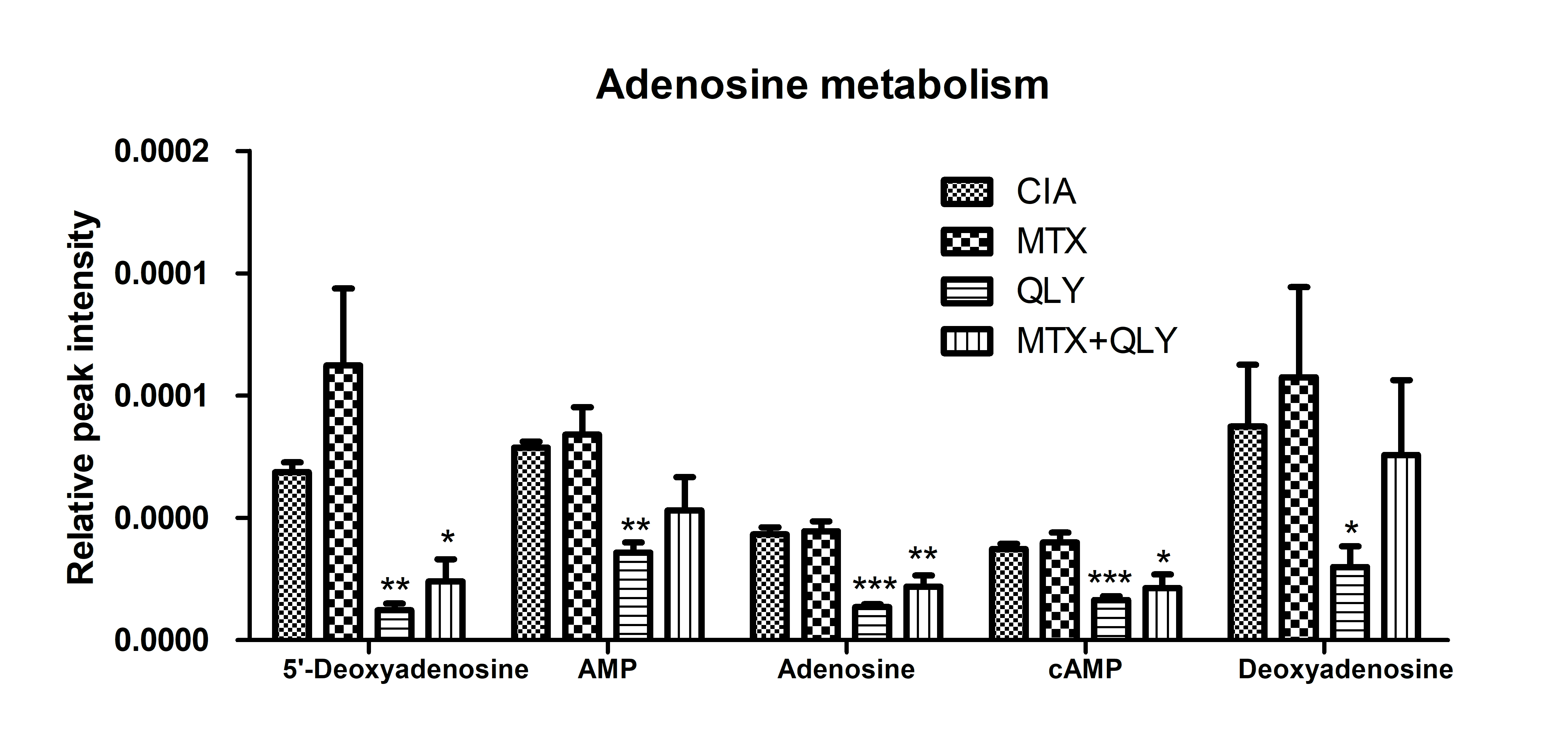

Supplement: FILE S1 — Ingredient information of QLY. [file Data_Sheet_1.ZIP › Supplementary/S13 Effects of QLY on adenosine metabolism in CIA rats..tif]

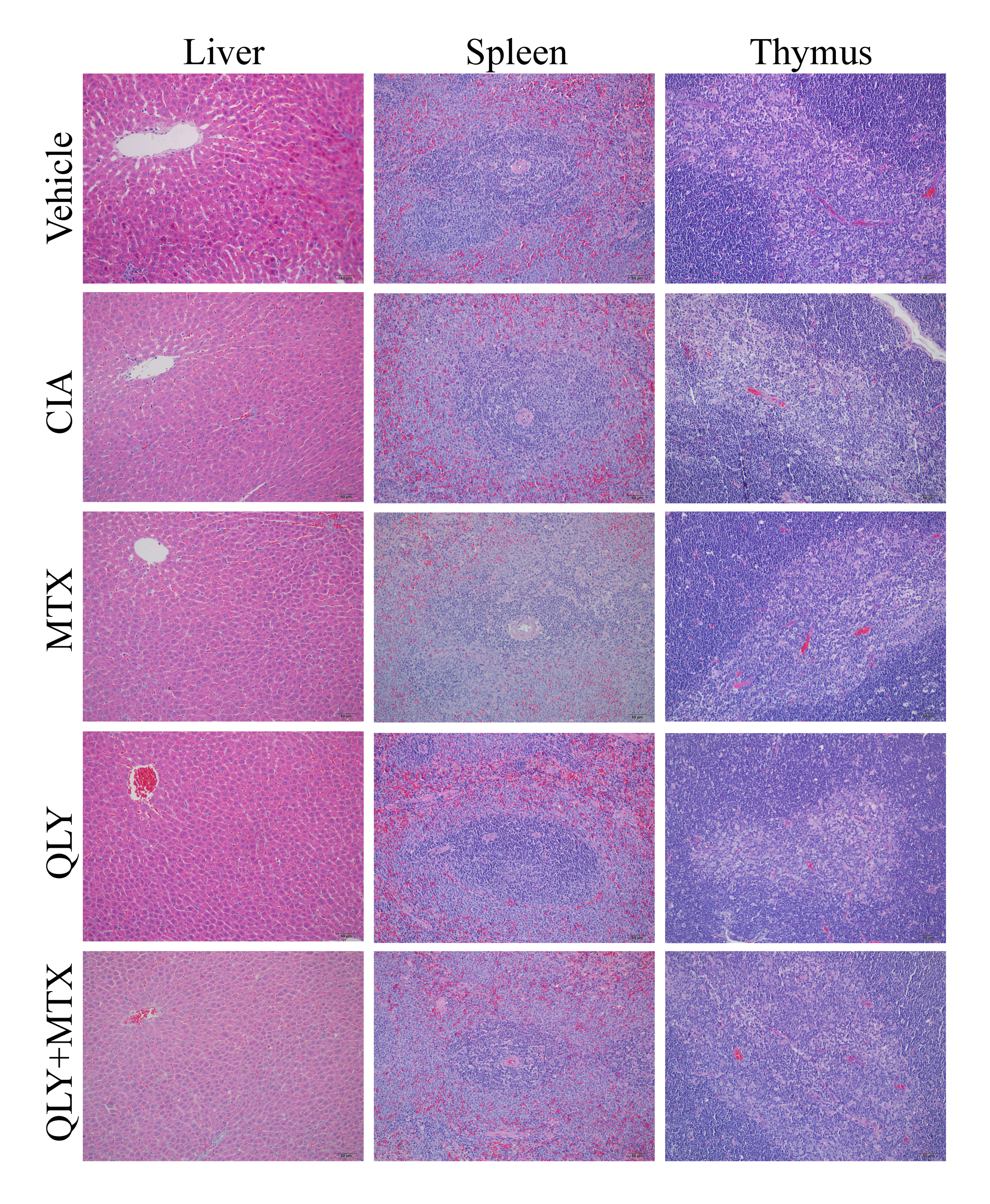

Supplement: FILE S1 — Ingredient information of QLY. [file Data_Sheet_1.ZIP › Supplementary/S4 Histological examinations of main organs of rats.tif]

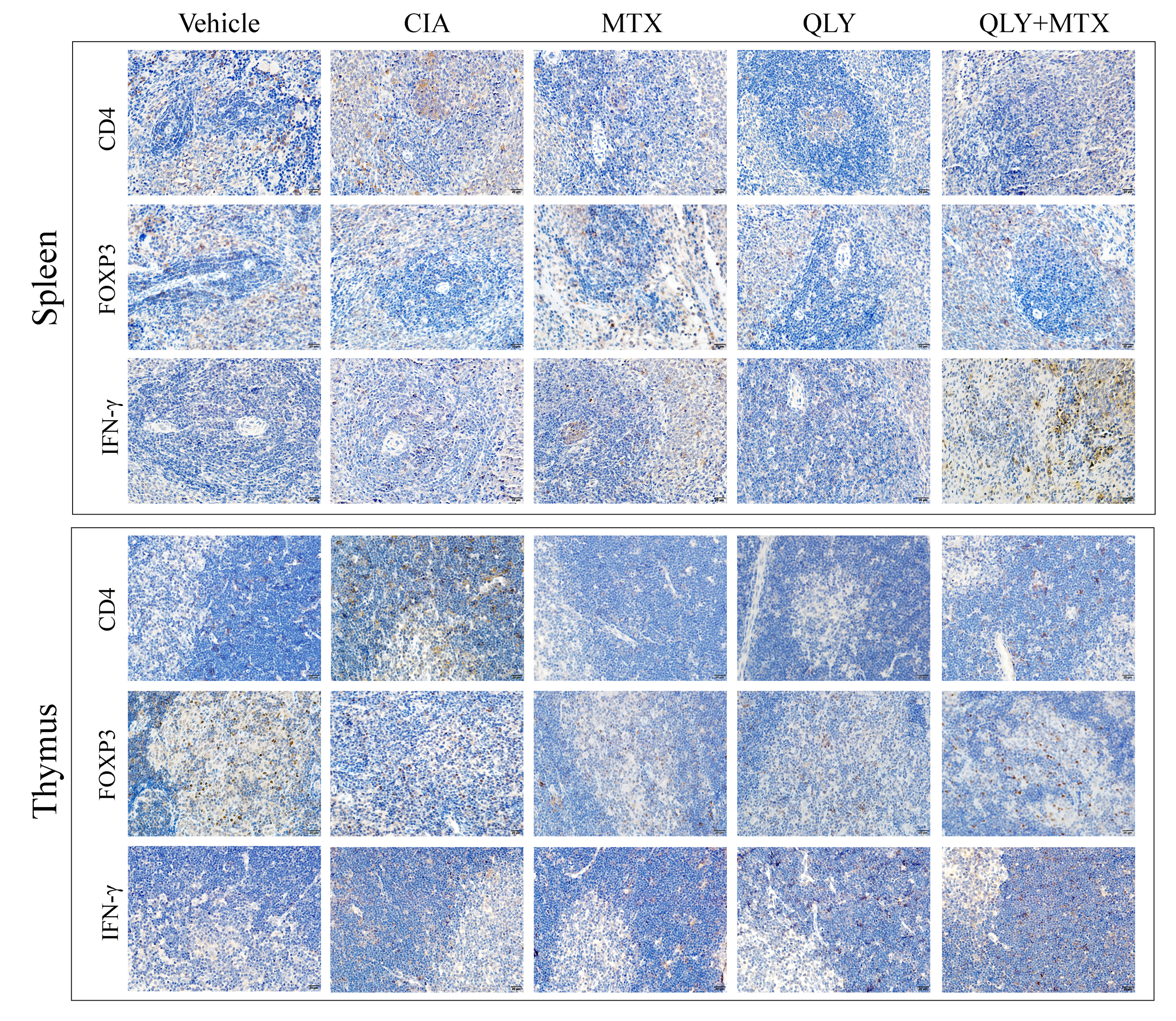

Supplement: FILE S1 — Ingredient information of QLY. [file Data_Sheet_1.ZIP › Supplementary/S5 Immunohistochemical examinations of spleen and thymus of rats.tif]

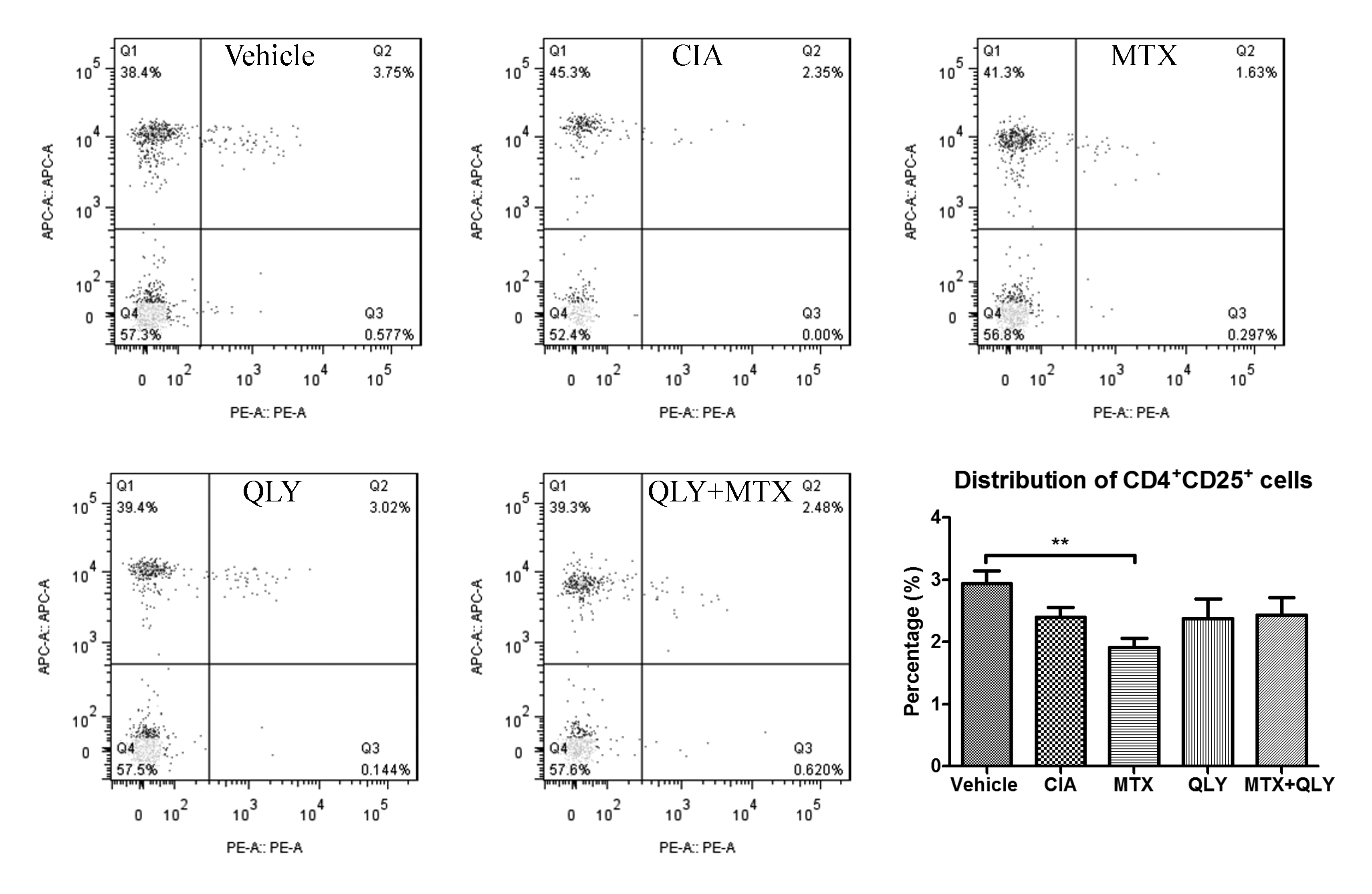

Supplement: FILE S1 — Ingredient information of QLY. [file Data_Sheet_1.ZIP › Supplementary/S6 Distribution of CD4+CD25+ cells in peripheral blood of rats.tif]
